# Revisiting Metformin: Annual Vitamin B12 Supplementation may become Mandatory with Long-Term Metformin Use

**DOI:** 10.4103/0975-1483.71621

**Published:** 2010

**Authors:** R Mahajan, K Gupta

**Affiliations:** *Departments of Pharmacology, Adesh Institute of Medical Sciences & Research, Bathinda – 151 109, Punjab, India*; 1 *Departments of Biochemistry, Adesh Institute of Medical Sciences & Research, Bathinda – 151 109, Punjab, India*

**Keywords:** Metformin, neuropathy, type 2 diabetes

## Abstract

Monitoring of adverse drug reactions of a drug is a continuous process and runs through-out the life of a drug. Many rare adverse effects of a drug are documented after years of use; when a single case (signal generation) is reported leading subsequently to reporting of more cases. Deficiency of Vitamin B12 (vit B_12_) is a known sequel of prolonged metformin therapy. It was recommended to have annual measurement of serum vit B_12_ levels in patients on long term metformin therapy way back in 1970 itself. After more than 50 years of use of metformin, we have come to know that metformin induced vit B_12_ deficiency can cause neuropathy; forcing to change the recommendation from annual screening of vit B_12_ levels to annual supplementation of vit B_12_.

## INTRODUCTION

Not all hazards of a drug are known before marketing. Some adverse effects of drugs come to fore only after years of use. That is why constant post-marketing monitoring is necessary and why clinicians are expected to report all adverse effects to the regulatory/monitoring agencies. A single case reported in the medical literature can also lead to the generation of a signal, and subsequently more cases are reported. This forms the core of pharmacovigilance.[1] In recent times, rare adverse effects due to long-term metformin therapy have been reported, thus necessitating revision of recommendations.

Metformin, a biguanide, was introduced in the United Kingdom in 1958, in Canada in 1972, and in the United States in 1995. It is the drug of first choice for the treatment of type 2 diabetes, particularly in overweight and obese people and those with normal kidney function.[2] As of 2009, metformin is one of the only two oral antidiabetics in the World Health Organization Model List of Essential Medicines.[3] Due to this, metformin, either used as monotherapy or as combination therapy with other oral antidiabetic agents or insulin, has become the most widely used antidiabetic drug. Due to widespread use, much is known about its side effects (SEs). The most dreaded SE of biguanides, lactic acidosis, is never a problem with judicious use of metformin.[4] Gastrointestinal SEs of metformin can be overcome by initiating metformin therapy at a low dose and slowly increasing the dose, by giving metformin after meals, or by utilizing a slow-release metformin preparation.[5]

## ADVERSE EFFECTS OF METFORMIN

One well-documented SE of metformin is malabsorption of vitamin B_12_(vit B_12_) and consequently low serum levels of the vitamin. Within the first 10–12 years after it came into use, it became evident that long-term metformin therapy causes vit B_12_malabsorption. Prevalence of vit B_12_malabsorption was found to be 30% in patients taking long-term metformin therapy, and low serum levels of vit B_12_were found in approximately 20% of patients having vit B_12_malabsorption.[6] In another study, low serum levels of vit B_12_ were reported in 17.5% of patients using 2 g of metformin daily for at least 2 years.[7]

The first case of vit B_12_ deficiency–induced megaloblastic anemia due to long-term metformin was reported in 1980. The patient developed megaloblastic anemia after 8 years of metformin use.[8] Subsequently, more cases were reported. In 2007, case of hyperhomocysteinemia-induced deep vein thrombosis was reported, where the hyperhomocysteinemia was apparently caused by long-term metformin therapy–induced vit B_12_ deficiency.[9] Recently, the first case of peripheral neuropathy due to metformin-induced vit B_12_ deficiency was reported.[10] So, what had been feared by early researchers in the field of metformin-induced malabsorption – that metformin can cause subacute combined degeneration of the cord, which can be easily mistaken as diabetic neuropathy – has proved true.[6,7]

## REVISTING METFORMIN

Annual measurement of serum vit B_12_ levels in patients on long-term metformin therapy was recommended way back in 1970s.[6–8] However, given the present setup of primary care centers in developing countries, with the lack of adequate laboratory facilities, it is doubtful that such monitoring will be possible in all diabetic patients.[11,12] In any case, this is hardly the routinely-followed method in patients on metformin therapy. Moreover, cost-effectiveness of annual measurement of vit B_12_ levels will also need to be considered, given that the incidence of type 2 diabetes is on the rise. Also, it must be remembered that vit B_12_ deficiency–induced neuropathy precedes the appearance of megaloblastic anemia. While the anemia of vitamin B_12_ deficiency is reversible, the progress of the neuropathy is only arrested and not reversed with initiation of vit B_12_ therapy. Even after measurement of low vit B_12_ levels, the differential diagnosis from other causes of vit B_12_ deficiency will be difficult to make. The fact that the symptoms of diabetic neuropathy resemble metformin-induced neuropathy will add to the confusion.

Thus, annual injections of vit B_12_ (in a dose of 1 mg) given to every patient on long-term metformin therapy will be a more practical and cost-effective method. This method will ensure replenishment of vit B_12_ stores for at least 1 year. It will also obviate the need for annual screening of vit B_12_ levels.
